# Epidemiology of Hip Dislocations in the United States From 1990 to 2019: A Temporal Study Using the Global Burden of Disease Database

**DOI:** 10.7759/cureus.86909

**Published:** 2025-06-28

**Authors:** Ambrose Loc T Ngo, Gabrielle Dykhouse, Taylor J Manes, Phillip C McKegg, Cameron J Sabet, Brett Barthman, Robert Golden

**Affiliations:** 1 College of Osteopathic Medicine, Kansas City University, Joplin, USA; 2 School of Medicine, Cornell University, Ithaca, USA; 3 Department of Orthopedic Surgery, OhioHealth Doctors Hospital, Columbus, USA; 4 Department of Orthopedic Surgery, Henry Ford Hospital, Detroit, USA; 5 Department of Surgery, Georgetown Medicine, Washington, DC, USA; 6 College of Osteopathic Medicine, A.T. Still University, Kirksville, USA; 7 Department of Orthopedic Surgery, George Washington University School of Medicine and Health Sciences, Washington D.C, USA

**Keywords:** bilateral hip dislocation, bone injury, hip joint in adults, orthopaedics trauma, traumatic hip dislocation

## Abstract

Introduction

Hip dislocations are devastating injuries that require urgent intervention to minimize the development of severe complications. This study aimed to evaluate the epidemiology of hip dislocations in the United States (U.S.) from 1990 to 2019.

Methods

This study is a descriptive retrospective epidemiological study. The Global Burden of Disease (GBD) database was used to collect epidemiological data on hip dislocation in the U.S. from 1990 to 2019. Data included years lived with disability (YLDs), prevalence, and incidence rates per 100,000 people. Data were regionally stratified into Northeast, Midwest, South, and West by the U.S. Census definition. Bartlett’s test was used to assess equal variance. Welch’s ANOVA was performed to assess regional differences to compare the means of different groups without assuming equal variances or equal sample sizes. The Games-Howell post hoc test was used to compare regions. Independent t-tests were performed to compare the means of each measure between males and females. Statistical significance was defined as p<0.05, and analyses were performed using IBM SPSS Statistics software, version 29.0.2.0 (IBM Corp., Armonk, NY).

Results

From 1990 to 2019, the U.S. saw a 1.67% decrease in the mean rate of YLDs, a 0.32% decrease in the mean prevalence rate, and a 4.74% decrease in the mean incidence rate of hip dislocations over the 29 years. Nationally, men experienced higher mean rates of YLDs, incidence, and prevalence compared to women, though only incidence was statistically significant (p<0.001). The Western region had the highest mean rates of YLDs, prevalence, and incidence rates of hip dislocation, while the Northeastern region experienced the lowest. Men had higher mean rates of YLDs in the Midwest (p=0.001), South (p<0.001), and West (p=0.004) regions. Men had a higher mean prevalence rate in the South (p=0.007), but not in other regions.

Conclusions

From 1990 to 2019, the U.S. experienced an overall drop in mean incidence, prevalence, and disease burden of hip dislocations, with men consistently showing higher rates across all measures compared to women. Regionally, the Western U.S. had the highest mean rates, while the Northeastern U.S. had the lowest. Our overall findings on the regional and sex-based disparities highlight the need for further targeted prevention strategies.

## Introduction

Dislocations of the hip are devastating orthopedic injuries that require urgent intervention. Among the elderly and frail population in the United States (U.S.), hip dislocations are the most common lower extremity joint dislocation [1]. According to Beebe et al., it is estimated that 46%-84% of all hip dislocations occur as a result of traffic accidents, traumatic falls, sporting injuries, and occupational injuries [2]. With hip dislocations, it is highly recommended to perform an anatomic reduction within six hours of injury to reduce the risk of developing avascular necrosis and post-traumatic osteoarthritis [3]. 

Patients with hip dislocations typically present with severe pain, immobility, and abnormal limb positioning. A flexed, abducted, and externally rotated hip points to an anterior dislocation, while a flexed, adducted, and internally rotated hip points to a posterior dislocation; the latter is more commonly encountered, comprising 90% of native cases [2, 4]. Furthermore, these injuries can be classified as simple or complicated. A simple dislocation is one without an associated fracture, whereas a complicated dislocation involves an associated fracture of the acetabulum, femoral head, or femoral neck [5]. Recognizing these characteristic presentations is critical, as they directly inform the urgency and method of intervention.

Additionally, hip dislocations may be treated non-operatively or operatively. Non-operative reduction of simple posterior dislocations involves techniques such as the Bigelow maneuver, in which longitudinal traction is applied with internal rotation of the hip [6]. Anterior dislocations involve longitudinal traction and external rotation, accompanied by pressure on the femoral head or pulling the femur laterally [7]. Pain management includes anti-inflammatories, physical therapy, or intra-articular steroid injections. However, complex dislocations or failed non-operative treatment of simple dislocations may require surgical interventions such as percutaneous fixation, open fixation, or replacement, including hemiarthroplasty or total hip arthroplasty (THA).

Regarding financial implications, Weber et al. found that patients admitted with a diagnosis of traumatic hip dislocation had increased lengths of stay and direct treatment costs as compared to other patients [8]. They experience added indirect costs of limited mobility and higher rehabilitation needs, which can also be seen following periprosthetic dislocations [8]. Furthermore, by 2034, it is estimated that the percentage of the U.S. population over 65 years of age will reach 25% [9]. Although the hip withstands force from multiple directions and recruits stability from many ligaments and muscles, these conforming forces begin to weaken and become unbalanced as a patient ages, predisposing them to hip dislocations [7]. Therefore, with a concurrent aging population, it may be expected that the prevalence of hip dislocations and their resulting economic burden will increase in the coming decades. However, the available information on temporal trends and geographical discrepancies of hip dislocations in the United States is limited. As such, this study aims to address that concern by offering a holistic national and regional assessment over 29 years.

## Materials and methods

Data sources and study design

This study is a retrospective descriptive epidemiological analysis of hip dislocation burden in the United States from 1990 to 2019. It was done utilizing the Global Burden of Disease (GBD) dataset that was developed by the Institute for Health Metrics and Evaluation [10]. The GBD dataset is comprised of the epidemiological data of 369 diseases and injuries across 204 countries and spans from 1990 to 2019. To provide estimates and projections, the GBD utilizes data from numerous primary and secondary sources, including administrative data, census data, demographic surveys, geospatial data, and modeled data. The methods and development of the GBD dataset have been described at length, and the disease burden estimates have been previously validated. Utilizing the U.S. Census Bureau definitions, the data were stratified into four regions: the Northeast, the Midwest, the South, and the West.

Outcomes

The outcomes of interest in this study included years lived with disability (YLD), incidence, and prevalence of hip dislocations. The World Health Organization defines a YLD as “one full year of healthy life lost due to disability or ill-health" [11]. Age-standardized rates of YLDs, prevalence, and incidence per 100,000 people were collected and utilized for both males and females in this study, consistent with the Institute for Health Metrics and Evaluation (IHME) methodology. These data were collected for the entire U.S. population as well as each state. Institutional review board approval was not obtained for this study as it does not contain any protected health information, and all data have been de-identified and are publicly accessible. As the GBD database uses statistical modeling to address gaps in data coverage, including predictive covariates and ensemble modeling techniques, no additional imputation was required for missing data in this study.

Statistical analysis

The statistical analysis in this study mirrors analytical methods used in previously published GBD analyses. To assess the potential need for multiple comparisons, an analysis of variance (ANOVA) of all measures was performed. Bartlett’s test was performed to assess the variance of the dataset and to evaluate whether this difference was equal or unequal. If unequal variability was found, a Welch’s ANOVA test was performed to assess the differences in regional rates of YLDs, prevalence, and incidence. A Games-Howell. The post-hoc test was also utilized to perform multiple comparisons between regions in regard to mean rates of YLDs, prevalence, and incidence if the variance was unequal. If variability was equal, Tukey’s post-hoc analysis was used. Then, an independent t-test was performed to compare the mean YLDs, prevalence, and incidence rates between males and females per region and amongst the entire U.S. Statistical significance was defined as p < 0.05. All statistical analyses were performed using IBM SPSS Statistics software, version 29 (IBM Corp., Armonk, NY).

## Results

National trends by gender analysis

**Figure 1:**
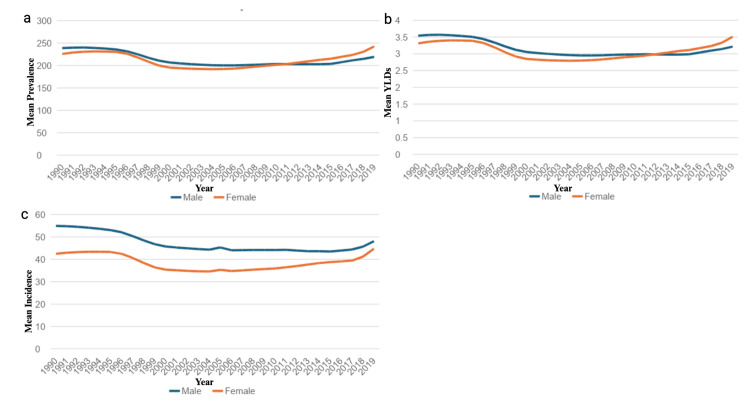
Prevalence, mean years lived with disability (YLDs), and incidence of hip dislocation by gender Figure 1a illustrates the mean age-standardized prevalence per 100,000 by sex over the same period. Figure 1b illustrates the mean age-standardized years lived with disability (YLDs) per 100,000 for males and females from 1990 to 2019. Figure 1c displays the mean age-standardized incidence per 100,000 by sex.

From 1990 to 2019, the United States saw a 1.67% decrease in the mean rate of YLDs, a 0.32% decrease in the mean prevalence rate, and a 4.74% decrease in the mean incidence rate of hip dislocations over the 29 years. For the entire United States, men had a higher mean rate of YLDs and a higher mean prevalence rate; however, this difference did not reach statistical significance (p > 0.05). Men also experienced a higher mean incidence rate compared to women (p<0.001) (Figures 1a-1c).

Regional difference analysis

**Figure 2:**
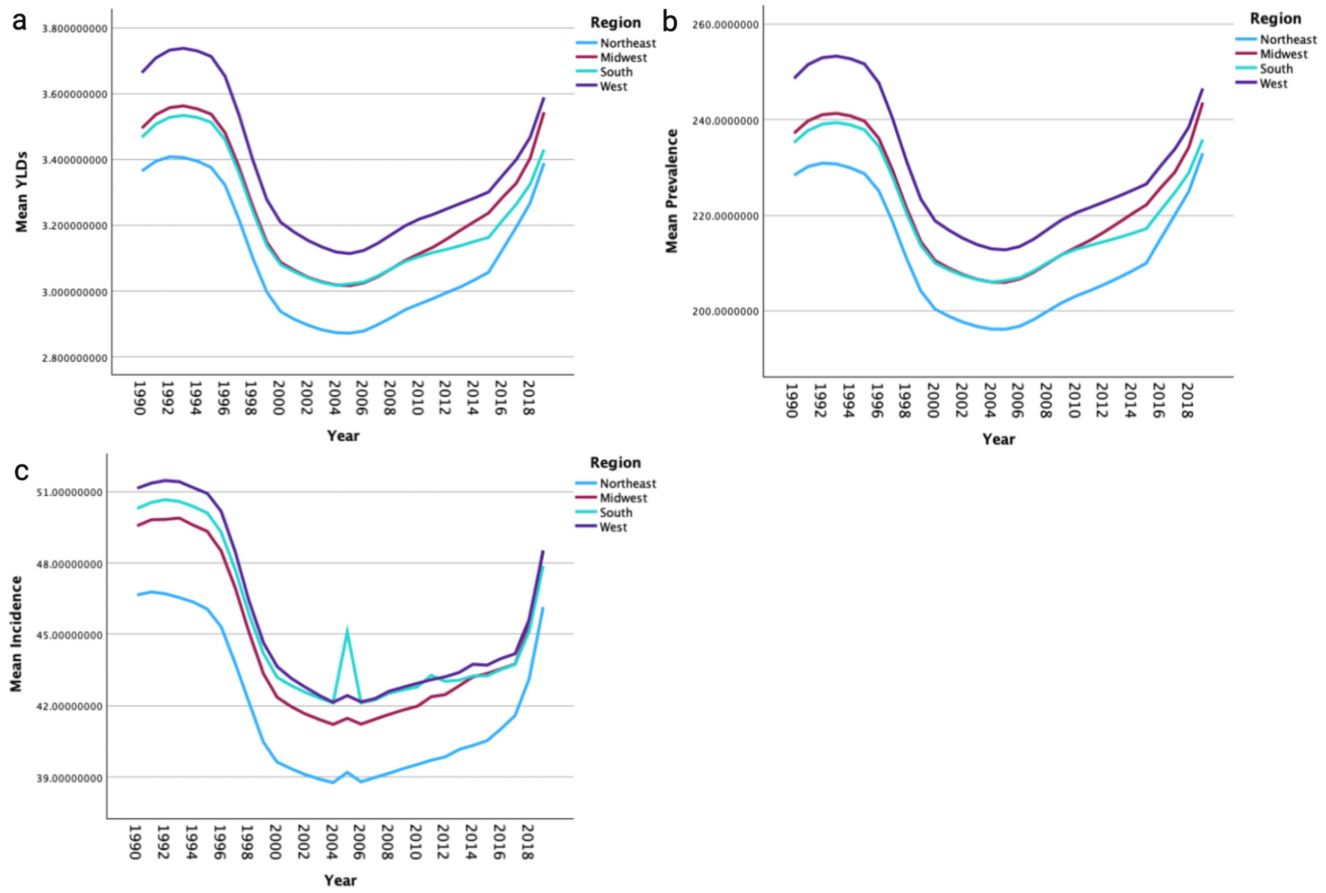
Comparison of mean years lived with disability (YLDs), prevalence, and incidence by region Figure 2a shows the mean age-standardized YLDs per 100,000 by region. Figure 2b presents the mean age-standardized prevalence per 100,000 by region. Figure 2c illustrates the mean age-standardized incidence per 100,000 by region.

Regional analysis demonstrated that the western region had the highest mean rate of YLDs, the highest mean prevalence rates, and the highest mean incidence rates compared to all other regions (Figures 2a-2c). The Northeastern region experienced the lowest mean rates of YLDs, prevalence, and incidence rates of hip dislocation. Men had higher mean rates of YLDs in the Midwest (p=0.001), South (p<0.001), and West (p=0.004) regions. Men had a higher mean prevalence rate of hip dislocations in the South (p=0.007); however, no significant difference was noted in the Northeast (p=0.442), Midwest (p=0.209), or West (p=0.233) regions. Men had a higher mean incidence rate compared to women in all regions (p<0.001).

State-level analysis

Illinois experienced the highest increase in mean rate of YLDs (18.01%) and incidence (15.03%), while New Hampshire experienced the highest increase in mean prevalence rate (20.02%) (Table 1). New Mexico experienced the greatest decrease in mean rates of YLDs (22.93%), while Alaska experienced the greatest decrease in mean prevalence (19.60%) and mean incidence rates (21.03%) of hip dislocations (Table 1).

**Table 1:** Percent change of disability-adjusted life years, years lived with disability (YLDs), prevalence, and incidence from 1990-2019

State	YLDs: 1990	YLDs: 2019	% Change	Prevalence: 1990	Prevalence: 2019	% Change	Incidence: 1990	Incidence: 2019	% Change
Alabama	3.6067	3.5008	-2.9353769	244.900589	225.085919	-8.0909035	52.8932213	48.9793621	-7.3995479
Alaska	4.4298	3.8771	-12.476916	300.999728	242.007171	-19.598874	64.539417	50.9682707	-21.027687
Arizona	3.6303	3.3359	-8.1100531	246.964669	227.383424	-7.9287636	50.0012609	45.7216625	-8.5589808
Arkansas	3.8160	3.3646	-11.831045	258.128427	235.731843	-8.6765275	57.5482381	47.1634497	-18.045363
California	3.3927	3.3342	-1.7240094	228.579816	242.377459	6.03624689	48.5813602	46.7144668	-3.8428184
Colorado	3.8942	3.2957	-15.368303	264.659234	230.672319	-12.841764	52.4567632	46.9494066	-10.498849
Connecticut	3.2936	3.1560	-4.1782662	223.165269	253.911145	13.777178	46.5447085	43.7803855	-5.9390703
Delaware	3.3551	3.6102	7.60307794	228.475656	238.618152	4.43920176	46.5234595	49.1779537	5.70571119
District of Columbia	3.3355	3.6649	9.87420865	225.801351	252.783864	11.9496687	47.7591699	50.3204571	5.36292234
Florida	3.4260	3.8490	12.3455976	232.612718	245.742001	5.64426722	47.6788679	51.3905293	7.78470957
Georgia	3.4992	3.3913	-3.0844966	237.835336	233.819664	-1.688425	50.6070854	47.5490112	-6.0427788
Hawaii	3.3223	3.7760	13.6554702	224.646747	238.55386	6.19065886	46.2357493	50.033788	8.21450671
Idaho	3.6278	3.7882	4.42035398	246.203462	261.961688	6.40048914	51.6129319	51.2479962	-0.7070626
Illinois	3.5241	4.1623	18.1083087	238.782108	252.872639	5.90099945	49.9773598	57.4921736	15.0364361
Indiana	3.3659	3.1430	-6.6222632	228.664392	251.570601	10.017392	48.9972819	48.1311407	-1.7677331
Iowa	3.4732	3.3802	-2.6780966	235.386708	225.61011	-4.1534196	47.725927	47.4856901	-0.5033677
Kansas	3.4300	3.6068	5.15329845	232.561708	260.724975	12.1100189	50.0576153	49.5574651	-0.999149
Kentucky	3.6343	3.2485	-10.614834	247.769802	244.157678	-1.4578548	52.213969	44.1727648	-15.400484
Louisiana	3.3128	3.3085	-0.1293177	225.036178	244.059908	8.45363183	49.9814484	48.8951055	-2.1734923
Maine	3.4922	3.7923	8.59299484	237.663502	225.59839	-5.0765526	47.3317929	46.9113385	-0.8883128
Maryland	3.4448	3.6601	6.25219531	233.344957	257.212935	10.2286236	50.2649269	50.7884752	1.04157786
Massachusetts	3.4030	3.3422	-1.7862188	230.948025	245.951703	6.49656016	46.545826	51.369828	10.363984
Michigan	3.1640	3.6286	14.6836987	214.832748	246.22799	14.6138063	45.2674617	41.1281067	-9.1442172
Minnesota	3.7872	3.8193	0.84695414	257.121508	237.016137	-7.8194049	51.5518971	44.9057238	-12.892199
Mississippi	3.5487	3.7582	5.90410779	239.536539	204.229922	-14.739554	52.6861013	51.01491	-3.1719775
Missouri	3.7431	2.9816	-20.343123	254.431645	232.224398	-8.7281781	52.8744211	48.8362751	-7.6372391
Montana	3.7209	3.5252	-5.2598641	253.088198	244.023415	-3.5816694	52.1543001	50.1237968	-3.8932616
Nebraska	3.5150	3.8193	8.6564687	238.520315	241.385715	1.20132327	50.2459369	47.0212376	-6.4178309
Nevada	3.3904	3.4507	1.77800405	230.128907	240.433894	4.47791942	47.7446587	47.9649186	0.46132879
New Hampshire	3.4934	3.5471	1.53953703	237.661814	285.243132	20.0205986	47.2954736	45.6423634	-3.4952821
New Jersey	3.0869	3.5745	15.7964545	209.365042	223.090031	6.55553029	44.2663107	47.3183937	6.89482132
New Mexico	3.8730	2.9849	-22.929799	263.399479	248.634177	-5.6056686	52.9522887	49.4907261	-6.5371352
New York	3.1728	3.4727	9.45341297	215.197255	251.644244	16.9365493	44.9619501	47.943568	6.63142473
North Carolina	3.4838	3.5419	1.66798774	236.081691	214.789254	-9.0190971	48.692165	48.7212092	0.05964873
North Dakota	3.3292	3.4782	4.47293778	225.984172	205.34004	-9.1352112	48.2112405	43.8487054	-9.0487925
Ohio	3.4708	3.6902	6.32347209	236.077996	229.831738	-2.645845	49.0455814	46.034991	-6.1383519
Oklahoma	3.5703	3.5908	0.57338498	242.582491	239.426354	-1.3010575	51.2365441	47.2574959	-7.7660354
Oregon	3.6540	3.2879	-10.017659	248.434565	261.781444	5.37239206	51.2786285	45.9002279	-10.488581
Pennsylvania	3.3417	3.5294	5.61645597	227.477938	240.878632	5.89098638	47.602317	48.585199	2.06477755
Rhode Island	3.4478	3.5106	1.82337666	233.883919	231.749701	-0.9125115	46.6692197	50.7366771	8.71550344
South Carolina	3.3907	3.3353	-1.63269	229.990152	227.282872	-1.1771289	49.3311799	45.1104617	-8.5558833
South Dakota	3.6180	3.3593	-7.14972	245.232828	247.991379	1.12487048	51.30734	50.3097652	-1.9443121
Tennessee	3.3978	3.3888	-0.2635283	230.863544	258.955304	12.1681227	50.5129599	53.696573	6.30256687
Texas	3.5942	3.6651	1.97238196	243.827202	253.349074	3.90517215	51.4777563	45.7812305	-11.065995
Utah	3.5355	3.4612	-2.1021561	239.82908	228.192934	-4.8518495	48.8372343	48.627606	-0.4292389
Vermont	3.7057	3.4294	-7.4573015	251.237017	217.562827	-13.403355	50.556291	48.5866397	-3.895957
Virginia	3.3490	3.4623	3.38075717	227.308988	265.656193	16.8700788	48.9960645	48.4551964	-1.1039013
Washington	3.6278	3.5180	-3.0264571	246.573442	233.251892	-5.4026702	49.1035981	47.4169124	-3.4349533
West Virginia	3.3776	3.2727	-3.1073789	229.359899	260.872972	13.7395738	49.031336	46.7810493	-4.5894868
Wisconsin	3.6962	3.6844	-0.3177017	251.462731	267.400693	6.33810133	51.9366022	42.0400387	-19.055085
Wyoming	3.7184	3.5780	-3.7734343	252.122062	230.136276	-8.7202943	52.8855711	48.3326062	-8.6090872

## Discussion

Based on the temporal trend, the findings suggest an improvement in prevention and management techniques for hip dislocations. The significant reduction in the incidence rate is likely due to advancements in diagnostic technologies and improved preventive measures in managing the condition. Clegg et al. stated that the use of multidetector CT, hip arthroscopy, and MRI has enormously influenced the understanding and management of hip dislocations, as they are now able to reveal parts of the anatomy and diagnostic information on displaced femoral heads that were not possible with conventional CT [4]. The impact of this improvement in technology may suggest an explanation for the reduction in the incidence and prevalence rates seen in the current study.

We found a significant gender difference where the mean YLDs, prevalence, and incidence rate of hip dislocation are higher for men than for women. This description had two important points. First, hip dislocation in men can be higher due to an increased exposure to high-energy trauma like motor vehicle accidents and contact sports. This is fairly consistent with previously published literature reporting that men had an elevated risk of hip dislocation. A study by Weber in 2022 reported that traumatic hip dislocations mostly affected males in high-energy mechanisms, including motor vehicle accidents and contact sports [8]. This echoes the results of the current study, where we observed that high-energy trauma mostly affected the high-risk male population; hence, this population needs more emphasis on preventive measures. Moreover, our findings parallel previous results from Gillinov and colleagues, who studied hip dislocations after total hip arthroplasty [12]. The team similarly found that even after surgery, younger age, female sex, and combined comorbidity indices signaled an increased risk of postoperative instability cases [12]. Reporting among 1,517 patients undergoing primary THA, the study’s authors found that 2.3% of patients experienced a dislocation within the first two years, of which 30% reported recurrence [12]. These results mirror the general trend of higher-risk groups, but the increased incidence rate among men in the present study also indicates that gender-specific factors might be involved in post-arthroplasty outcomes through different mechanisms.

In the regional analysis, the mean rates of YLDs, prevalence, and incidence of hip dislocation were highest in the South, and those were lowest in the Northeastern region of the US. Specifically, males in the South have the highest incidence rate of all four regions, with a rate of 50.14 per 100,000. For females, the West leads with 41.15 per 100,000. Some of these differences can be explained by the demographics and lifestyles associated with these regions. For example, favorable weather found in the South and West has been associated with higher physical activity times among older adults, which may be contributing to the higher incidence of hip dislocations in these regions [13]. The overall higher rate among men could be explained by the fact that males are more likely than females to play high-level sports, perform hard manual labor, and engage in other high-risk leisure activities that lead to trauma [14-16].

Notably, our study revealed that Illinois had the highest increase in mean rates of YLDs (18.01%) and incidence (15.03%). Potentially contributing to this rise is the fact that the state’s fastest-growing population is the group aged 65 or above, rising by 33.9% between 2010 and 2022 [17]. Beyond age, traumatic injuries like automobile accidents may also be contributing to this state’s growth in YLDs and incidence. Thus, we suggest that the Illinois Department of Transportation further investigate statewide data on automobile accidents to identify modifiable risk factors and redesign physical roadways and their laws as needed [18].

In contrast to Illinois, New Mexico had the largest decrease in YLDs (22.93%), and Alaska had the largest decrease in prevalence (19.6%) and incidence (21.03%). The reduction in hip dislocation injuries in New Mexico could be due to the 2006 implementation of the statewide Department of Transportation Highway Safety Improvement Program (HSIP), using a data-driven approach to improve safety on our roads and reduce injury and fatal crashes on our state roadway system [19]. Another important contributor in New Mexico to these improvements has been Safer New Mexico Now’s programs that provide public education, as well as enhanced law enforcement efforts such as Click It or Ticket that reduced alcohol-related crashes and increased seat belt use [20].​ Alaska has similarly enjoyed a decline in hip dislocations, likely due in part to educational online programming from its statewide Senior Fall Prevention program, from its Commission on Aging [21].

Additionally, hip dislocations were observed to decrease drastically over the investigated timeframe. A decrease in hip dislocations can be attributed to advances in diagnostic technologies, such as multidetector CTs, hip arthroscopies, and MRIs [22]. This, as a result, indicates that advances in prevention and management strategies are effective. 

Furthermore, regional variations in hip dislocation rates reflect possible demographics, lifestyle, and environmental influences. Compared to the Northeast, the South had the highest mean rate of hip dislocation, which emphasizes the need to alternate interventions based on regional characteristics. Finally, the rate of hip dislocations in Illinois had notable increases, whereas it decreased significantly in areas such as New Mexico and Alaska. This suggests the impact of policy and program interventions. As mentioned earlier, it is estimated that by 2034, 25% of the U.S. population will be over the age of 65 [9]. Therefore, the need to continue efforts to improve preventative measures and diagnostic tools is essential so that hip dislocations can be mitigated as much as possible. 

This study has several limitations concerning the modeled data used from the GBD database. A key limitation of this study is the lack of data from 2019 onwards during our data collection and analysis process. This lack of access could limit our evaluation of recent patterns and may affect the applicability of the results to contemporary situations. Additionally, input measurements such as coding practices would influence over time changes, while reporting gaps for specific injuries could impact some estimates. Data discrepancies across regions may reflect different infrastructure and healthcare access or even true differences in reporting and diagnosis capacity instead of actual incidence variation. In addition, the ecological nature of the data restricts control for confounding factors like socioeconomic status, comorbidities, and injury mechanisms beyond broad groupings. Addressing these trends requires understanding their individual-level risk factors through prospective cohort studies. Moreover, intervention trials at the regional level combined with statewide systems for monitoring hip dislocation injuries may evaluate public health impacts and discover changeable determinants postulated by them.

Overall, this study presents a critical sex- and region-based disparity that informs resource allocation planning alongside targeted prevention, exposing modifiable determinants, which is novel in the longitudinal epidemiological review of hip dislocations in the U.S.

## Conclusions

Overall, this detailed examination of hip dislocation occurrences in the U.S. between 1990 and 2019 has given us insights into its regional spread and related factors. Our study highlighted gender differences in incidence, prevalence, and YLDs of hip dislocations, consistently showing higher rates among men. This overall underlines the necessity for targeted measures for at-risk male groups involved in activities like high-impact sports or vehicle accidents. By understanding the trends in hip dislocations and associated injuries, we can enhance technology and treatment methods. We hope our findings guide future improvements in the prevention of injury and the development of diagnostic tools to improve the treatment of hip dislocations. Indeed, such measures can be most effective through concerted intervention aimed at at-risk populations residing in the regions with the highest incidence and prevalence rates. 
